# Silicon Thermo-Optic Switches with Graphene Heaters Operating at Mid-Infrared Waveband

**DOI:** 10.3390/nano12071083

**Published:** 2022-03-25

**Authors:** Chuyu Zhong, Zhibin Zhang, Hui Ma, Maoliang Wei, Yuting Ye, Jianghong Wu, Bo Tang, Peng Zhang, Ruonan Liu, Junying Li, Lan Li, Xiaoyong Hu, Kaihui Liu, Hongtao Lin

**Affiliations:** 1 State Key Laboratory of Modern Optical Instrumentation, College of Information Science and Electronic Engineering, Zhejiang University, Hangzhou 310027, China; zhongchuyu@zju.edu.cn (C.Z.); atticusmhh@zju.edu.cn (H.M.); ml_wei@zju.edu.cn (M.W.); junyingli@zju.edu.cn (J.L.); 2 State Key Laboratory for Mesoscopic Physics, Frontiers Science Center for Nano-Optoelectronics, School of Physics, Peking University, Beijing 100871, China; zhibinzhang@pku.edu.cn (Z.Z.); xiaoyonghu@pku.edu.cn (X.H.); khliu@pku.edu.cn (K.L.); 3 Key Laboratory of 3D Micro/Nano Fabrication and Characterization of Zhejiang Province, School of Engineering, Westlake University, Hangzhou 310024, China; yeyuting@westlake.edu.cn (Y.Y.); wujianghong@westlake.edu.cn (J.W.); lilan@westlake.edu.cn (L.L.); 4 Institute of Advanced Technology, Westlake Institute for Advanced Study, Hangzhou 310024, China; 5 Institute of Microelectronics of the Chinese Academy of Sciences, Beijing 100029, China; tangbo@ime.ac.cn (B.T.); zhangpeng1@ime.ac.cn (P.Z.); liuruonan@ime.ac.cn (R.L.)

**Keywords:** mid-infrared, 2 μm waveband, thermo-optic switch, graphene heater

## Abstract

The mid-infrared (MIR, 2–20 μm) waveband is of great interest for integrated photonics in many applications such as on-chip spectroscopic chemical sensing, and optical communication. Thermo-optic switches are essential to large-scale integrated photonic circuits at MIR wavebands. However, current technologies require a thick cladding layer, high driving voltages or may introduce high losses in MIR wavelengths, limiting the performance. This paper has demonstrated thermo-optic (TO) switches operating at 2 μm by integrating graphene onto silicon-on-insulator (SOI) structures. The remarkable thermal and optical properties of graphene make it an excellent heater material platform. The lower loss of graphene at MIR wavelength can reduce the required cladding thickness for the thermo-optics phase shifter from micrometers to tens of nanometers, resulting in a lower driving voltage and power consumption. The modulation efficiency of the microring resonator (MRR) switch was 0.11 nm/mW. The power consumption for 8-dB extinction ratio was 5.18 mW (0.8 V modulation voltage), and the rise/fall time was 3.72/3.96 μs. Furthermore, we demonstrated a 2 × 2 Mach-Zehnder interferometer (MZI) TO switch with a high extinction ratio of more than 27 dB and a switching rise/fall time of 4.92/4.97 μs. A comprehensive analysis of the device performance affected by the device structure and the graphene Fermi level was also performed. The theoretical figure of merit (2.644 mW^−1^μs^−1^) of graphene heaters is three orders of magnitude higher than that of metal heaters. Such results indicate graphene is an exceptional nanomaterial for future MIR optical interconnects.

## 1. Introduction

Mid-infrared (2–20 μm spectral range [1]) is a practically important waveband covering a broad range of wavelengths with significant applications [2], including chemical [3], gas [4], and biological [5] sensing, imaging [6], infrared countermeasures [7], free-space communications [8], wind trace detection [9] and precise surgery [10]. So far, most of the mid-infrared systems are based on benchtop instruments. Integrating critical components at the chip level [11,12] would be an indispensable choice to achieve higher reliability, lower cost, smaller footprint, and reduced power consumption [1]. Laser sources [13], amplifiers [14], low-loss waveguides [15], wavelength gratings [16], multiplexers [17], power splitters [18], resonators [15], switches [19,20,21], photodetectors [22], and other components for the integrated photonic circuit at MIR waveband have already been widely studied for lab-on-chip applications.

Among the on-chip photonic components, optical switches [23] (electro-optic and thermo-optic type) work for wavelength tuning, light intensity modulating, and optical path switching. They are critical in optical phased array [24,25], optical sensing [26], photonic computation [27], and optical routing [28], etc. Electro-optic switches exhibit ultra-high-speed but suffer from high optical loss and large device size [29]. The thermo-optic switches show advantages in high tuning efficiency, compact footprint, and low insertion loss [30]. Most of the thermo-optic switches demonstrated so far were working at the telecommunication window. The emerging mid-infrared applications require fast and energy-efficient TO switches in mid-infrared waveband. High heating efficiency, low-loss of the heaters for the phase-shifting are significant for high-performance mid-infrared TO switch. Conventional micro-heaters of TO switches include metallic heaters and doped-silicon heaters. Metallic heaters [31,32,33] were widely utilized for TO switches, but the parasitic optical absorption from the metal required the heaters to be placed away from the waveguide core. Thus, the heating efficiency would be low, and it becomes even severe for devices operating at a longer wavelength. Doped-Si-heaters [34,35,36] generate Joule heat directly inside the waveguides leading to high-efficient heating. However, the free-carrier absorption of doped silicon can increase to be hundreds of dB/cm at a longer wavelength in MIR waveband [37,38,39,40], which makes it unfavorable for low loss mid-infrared TO switch.

Two-dimensional materials [41,42] own remarkable optical and electrical properties and can be alternative heating materials for TO switch, which are favorable for photonic integrated circuits at MIR waveband [43]. Graphene is an exceptional heater candidate that offers extraordinary physical properties [44,45,46], including ultra-wide working waveband from visible light to microwave [47] and high thermal conductivity [48]. Most importantly, the absorption of graphene can be tuned by dope-engineering [49]. The propagation loss of the MIR light can be reduced to virtually zero, making it suitable for long-wavelength operation. Graphene heaters have been integrated into polymer waveguides [50,51], which can achieve low π phase-shift power consumption (*P_π_*) of 0.39 mW. However, the low thermal conductivity of polymer leads to a long response time of 92.4 μs, and polymers also become opaque in the mid-infrared waveband. The figure-of-merit (*FOM*) of a TO device combines π-phase-shift power consumption and response time τ in the form of 1/(*P_π_·τ*), which could be used to evaluate the performance of devices based on different materials and devices platforms [52]. Recently, graphene has also been integrated with inorganic photonic structures with higher thermal conductivities to fabricate TO switches operated in the telewavelength window with much faster response [48,53]. To further reduce the power consumption without slowing down the response time, graphene heaters could be integrated with resonators [54] or photonic crystal structures [55,56]. Silicon microring TO switches with graphene heaters covering the microring part achieved superior performance with a switching time of 3.6 μs and a *P_π_* of 9 mW, respectively [57]. Taking advantage of the slow light effect, silicon photonic crystal waveguide TO switches with monolayer graphene heaters on top exhibited an unparalleled switching time of 0.75 μs [55]. Graphene heater can also be integrated into chalcogenide photonics [58]. Chalcogenide photonic-crystal cavity with a graphene heater embedded into the center realized unprecedented energy efficiency of 7.6 nm/mW [56]. Thus far, graphene TO switches could achieve sub-milliwatt switching power, microsecond-level response time, and extinction ratios as high as 30 dB at the telewavelength range. Moreover, through tuning the Fermi level of graphene, it could be transparent in the mid-infrared waveband. Graphene would be an ideal materials platform for mid-infrared thermo-optic switches, but no work has been reported yet.

In this context, we demonstrated the first silicon TO switch with graphene heaters operating at 2-μm mid-infrared waveband, which is also considered a new communication window for the next-generation optical communication [59] to meet the urgent demand for expanding the bandwidth capacity [60,61]. We fabricated two types of graphene-based TO devices based on 220 nm silicon-on-insulator (SOI). Microring resonators (MRR)-based TO switches were designed for light signal modulation, and 2 × 2 Mach-Zehnder interferometer (MZI) TO switches were designed for lightpath routing. The MRR-based TO switch achieved a fast 10–90% rise time of 3.72 μs, *P_π_* power consumption of 14.42 mW, and comparable *FOM* of 0.0175 mW^−1^μs^−^^1^ to previously reported silicon TO switches with graphene heaters. Our MZI device showed a high modulation depth of 27.8 dB. Lastly, a complete numerical analysis of the device’s performance was demonstrated. The design strategy of the graphene heater and the performance limits was given. Optimal structural parameters and graphene with a Fermi-level of 0.43 eV for the MRR-based TO switch could achieve an ultrahigh *FOM* of 2.644 mW^−1^μs^−1^. 

## 2. Device Fabrication and Characterization Method

**Figure 1:**
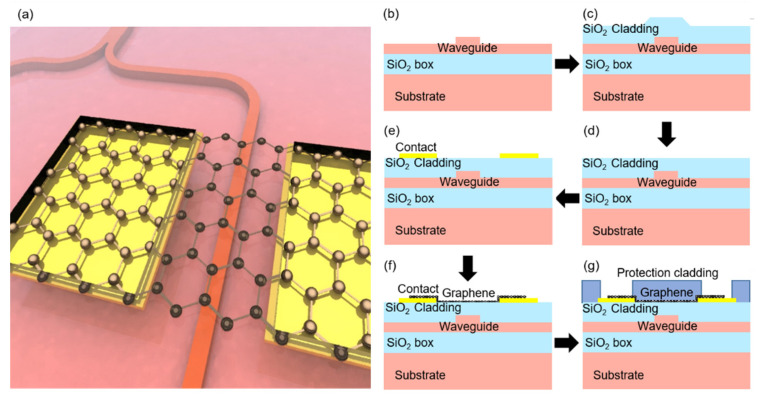
(**a**) Three-dimensional illustration of the MZI-based TO switch with the graphene heater. (**b**–**g**) Brief process flow of the TO switch with graphene heaters. (**b**) Waveguide fabrication. (**c**) SiO_2_ deposition as a cladding layer. (**d**) Chemical-mechanical planarization of the cladding. (**e**) Electrode contact fabrication. (**f**) Graphene transfer and patterning. (**g**) Protection cladding coating with electrode contact windows opened.

The schematic illustration of the MZI device is shown in Figure 1a. Our devices were based on the SOI platform. As illustrated in Figure 1g, the graphene was coated above the ridge waveguide with flattened silica cladding. The brief process flow is demonstrated in Figure 1b–g. Our devices were fabricated on an SOI wafer with a 220-nm-thick device layer, and a 2-µm-thick SiO_2_ box layer by multi-project-wafer (MPW) involved processes. First, the waveguide structures were patterned by deep ultra-violet photolithography and etched by inductive coupling plasma (ICP) process to a ridge depth of 150 nm and width of 600 nm. Next, a layer of 1-µm-thick SiO_2_ cladding was deposited by plasma-enhanced chemical vapor deposition (PECVD). Then the silica cladding was polished by chemical mechanical polishing (CMP) to a thickness of about 300 nm (150 nm higher than the waveguides). The titanium/gold (Ti/Au, 5 nm/100 nm) contact electrodes were patterned by electron beam lithography (EBL) and deposited using electron beam evaporation. The contact distances were 2.6/7 μm for the MRR/MZI-based switches, preventing the excessive absorption from metal contacts. Then the chemical-vapor-deposition (CVD) graphene [62] was transferred onto the samples by the wet-transfer method [63]. The loaded graphene was patterned by i-line photolithography, and the unwanted part was etched using oxygen plasma. Finally, a layer of polymer protection cladding was coated, and contact windows were opened for characterization. By these approaches, two types (MRR-type and MZI-type) TO switches were fabricated.

**Figure 2:**
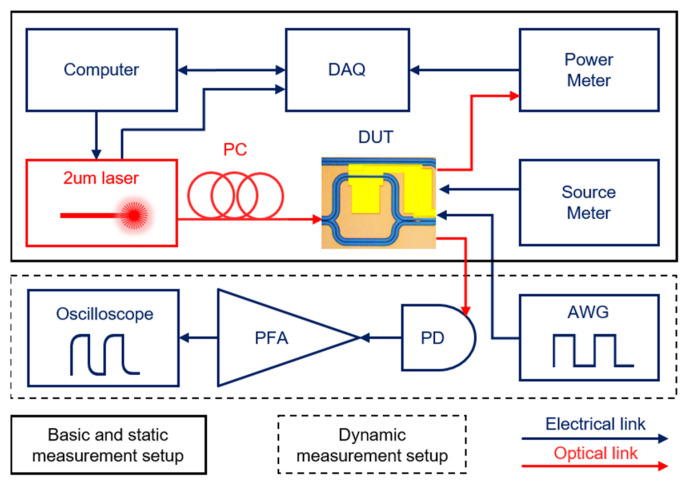
Schematic of the measurement setup. DAQ: data acquisition equipment, PC: polarization controller, DUT: device under test, AWG: arbitrary waveform generator, PD: photodetector, PFA: pulse forming amplifier (based on photomultiplier tube).

The measurement setup is shown in Figure 2, where a fiber coupling system [36] (not drawn in the figure) is used for light coupling of the devices. A computer was used for controlling the tunable 2-μm laser (Newfocus TLB-6700) for wavelength sweeping and processing the data stream from the data acquisition equipment (DAQ, NI USB-6212), which was used for data synchronization between the laser and the power meter. A polarization controller (PC) was used to adjust the polarization of the input light to optimize the coupling efficiency. The transmitted optical signal was read by the power meter. A source meter was used for static measurement to apply voltage and monitor the current flowing through the graphene heaters. The peak shift of the devices and I-V/O-P (current-voltage/transmission-power) curves could be obtained. An arbitrary waveform generator (AWG, Siglent SDG6032X-E) was utilized to apply the modulation voltage for dynamic characterization. The device’s optical output was connected to a photodetector, while its photocurrent was amplified by a pulse forming amplifier (PFA). The amplified modulated signal was recorded by an oscilloscope (Siglent SDS5104X), and our devices’ response time could be obtained.

## 3. Results and Discussion

### 3.1. MRR-Based Switches

The MRR-type switches play an essential role in silicon photonics. The MRR can behave as a spectral filter, which can be used for applications such as wavelength division multiplexing and sensing. The position and the shape of the dips are very sensitive to structural parameters. In most cases, the spectrum of the fabricated device could not be predicted precisely because of the fabrication error. Therefore, wavelength tuning is necessary for wavelength-relevant applications, and thermally modulating the refractive index of the waveguide in the microring resonator is a straightforward and effective approach. The switching function of an MRR works in the same principle [64].

**Figure 3:**
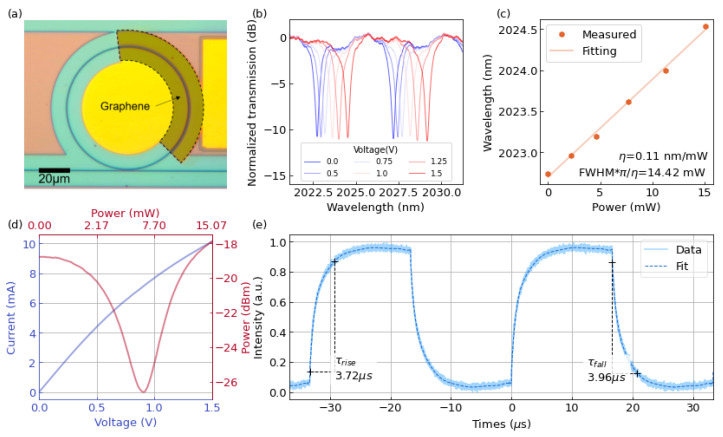
(**a**) Microscope image of an MRR-based graphene TO switch. The graphene is in a fan-shaped area. (**b**) Normalized transmission spectra of the switch under different applied voltages. (**c**) Resonant peak shift of the switch and fitting. (**d**) I-V feature of the graphene heater and O-P curve of the switch. (**e**) Time response of the MRR-based TO switch. Three-dimensional illustration of the MZI-based TO switch with the graphene heater.

We fabricated MRR-based switches with a ring radius of 40 μm and a graphene length of 100 μm. The microscope image of the MRR-based TO switch is displayed in Figure 3a, where the graphene shows a fan-shaped pattern. The coated waveguide area will be heated up when applying the driving voltage to the graphene heater. This would increase the refractive index and consequently brings in a shift of resonance wavelength.

The resonance wavelengths of measured transmission spectra were red-shifted, as depicted in Figure 3b. The energy efficiency *η* of the switch was calculated to be 0.11 nm/mW by fitting the curve in Figure 3c. The switch claims a *P_π_* of 14.42 mW, where *P_π_* of a cavity-type switch is defined as the power consumption to detune the resonator by a phase shift with a product of *π* and the full width at half maximum (*FWHM*) of the resonance peak [52]. It should be noted that, the minimum electric power to achieve decent modulation effect, i.e., extinction ratio of 8 dB, is only about 5.18 mW (0.8 V). The Q factor of the ring resonator under test was about 4 × 10^3^ and the *FWHM* was 0.505 nm. By decreasing the propagation loss from the waveguide (1.62 dB/cm) [65] and absorption loss from graphene (calculated to be 132 dB/cm), the Q factor of ring resonators could be improved, and the *P_π_* power consumption can be further suppressed. This could be improved by optimizing the fabrication process to reduce the scattering loss of the waveguide and doping graphene to ensure less absorption. The sheet resistance of the heater is calculated to be about 125 Ω from the current-voltage (I-V) curve shown in Figure 3d. Moreover, the optical transmission-power (O-P) characterization exhibited an extinction ratio of about 9 dB. As depicted in Figure 3e, the switch’s 10–90% rise and 90–10% fall time are 3.72 μs and 3.96 μs, respectively, via applying a 0.8 V square-wave driven signal with a frequency of 30 kHz and a duty cycle of 0.5. The switch realized a *FOM* of 0.0175 mW^−1^μs^−1^, which is the highest performance among the reported graphene-assisted silicon TO devices operating at the 2 μm waveband.

### 3.2. 2 × 2 MZI Switches

The MZI-type 2 × 2 optical switch is also one of the indispensable components in photonic integrated circuits, which is conventionally constructed by two 3-dB splitters/couplers using multimode interference (MMIs) and connecting waveguides as the two MZI arms. By tuning the optical phase difference of the light between two arms of the MZI, the optical intensity distribution of the output ports can be modulated, realizing optical switching for applications including cross-connection, optical routing, and information processing.

**Figure 4:**
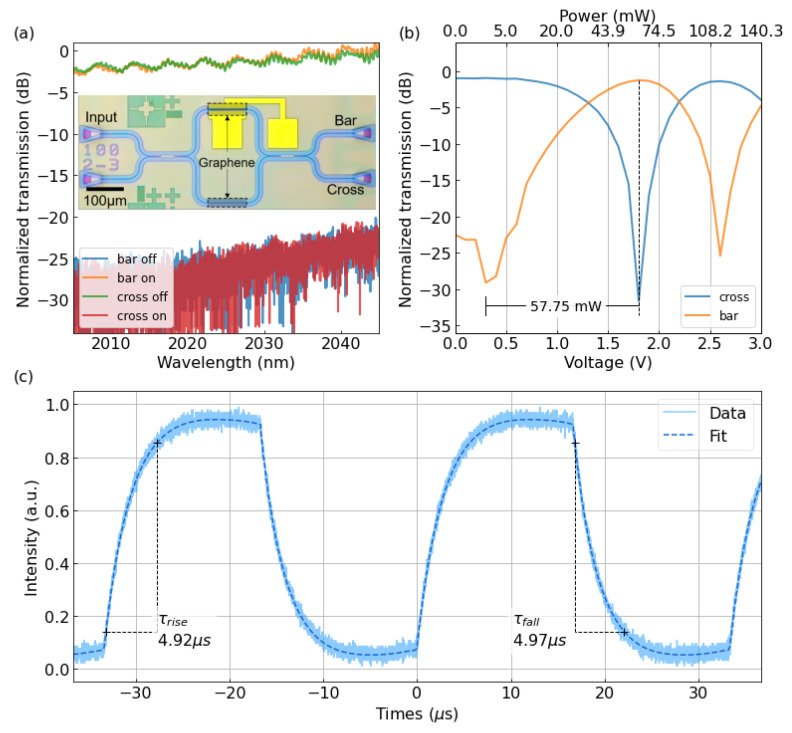
(**a**) Normalized transmission at the bar- and cross-ports of the 2 × 2 MZI switch (including the loss of grating couplers). Inset is the microscope image of the switch. (**b**) Normalized transmission of different ports under varying applied voltages and the corresponding electrical power. (**c**) Time response of the MZI-based 2-μm-waveband TO switch.

We fabricated balanced 2 × 2 MZI TO switches with 100-μm-long graphene on both arms of the MZI to balance the loss difference between the two arms. The microscope view of the switch is shown in the inset of Figure 4a. The transmission spectra of the device without/with a 1.8 V driving voltage indicated an insertion-loss of about 2 dB and a maximum on-off ratio of 27.08 dB, as shown in Figure 4a. The insertion loss was mainly induced by the absorption of graphene and the insertion loss from MMI (0.35 dB per MMI), and it could be minimized by doping the graphene, which will be discussed later. Through tuning the voltage applied across the graphene, the temperature rising of the waveguide varied, and the phase difference between the two arms changed.

The light from two arms of the MZI with variable phase delay was combined by a 2 × 2 MMI, resulting in different power distribution at the output ports. Figure 4b demonstrates the measured O-P curve, indicating a *P_π_* of 57.75 mW. Such high-power consumption was due to an extensively long contact distance which will be explained later.

The dynamic response of the switch was also characterized and demonstrated in Figure 4c. By applying a square-wave signal with a voltage amplitude of 1.8 V, a frequency of 30 kHz, and a duty cycle of 50%, the MZI TO switch realized a 10–90% rise time of 4.92 μs and a 90–10% fall time of 4.97 μs. Thus, the *FOM* of the switch is calculated to be 0.003 mW^−1^μs^−1^.

## 4. Numerical Analysis and Discussion

Table 1 lists the performance of some silicon TO modulators or switches with different kinds of heaters including the prediction of graphene-based MRR TO switch. Compared with metallic heaters, graphene could be used as transparent heaters, and is not necessary to be placed microns away from the waveguide to prevent parasitic optical loss. Thus, graphene-based TO switch could achieve a shorter response time and gain superior energy efficiency benefiting from the smaller thermal mass. Our MRR TO switch is the first achieved mid-infrared graphene-based TO switch with *FOM* comparable with TO switches using doped-silicon heaters. Although the doped-silicon TO modulators show better performance at present, our theoretical prediction implies that graphene is a more appropriate heater material at MIR waveband, which will be explained in the following session.

**Figure 5:**
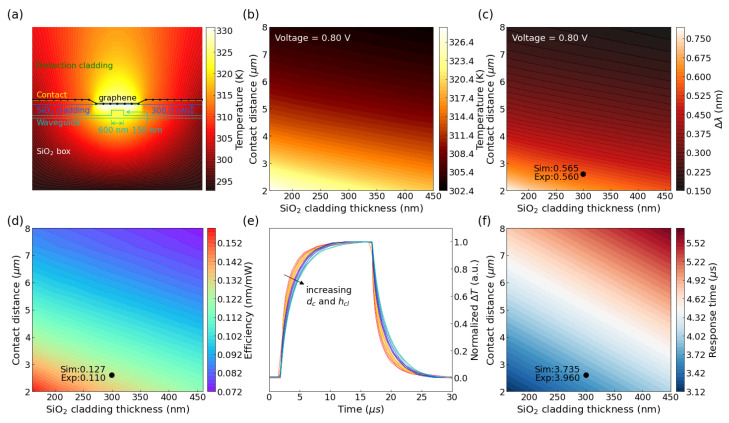
Simulation of the graphene-on-SOI structure. (**a**) Schematic cross-section and simulated temperature distribution of the graphene-on-SOI phase shifter with a contact distance of 2.6 μm under a driving voltage of 0.8 V. (**b**) Temperature mapping and (**c**) wavelength shift with different contact distances (*h_cl_*) and cladding thicknesses (*d_c_*) at 0.8 V. (**d**) Heating efficiency *η* of the device with different *h_cl_* and *d_c_*. (**e**) Dynamic heating progress of the waveguide with different *h_cl_* and *d_c_*. (**f**) Response time mapping with *h_cl_* and *d_c_*. Simulated (Sim) and experimental (Exp) values of our devices are labelled in (**c**,**d**), and (**f**) with structural points marked by circles.

To investigate the theoretical performance limits of graphene-based TO-switches, optical modeling of the waveguide modes and finite element method thermal simulations of the graphene phase shifter were carried out using Lumerical MODE solution and the COMSOL Multiphysics package, respectively. Figure 5a illustrates the temperature distribution in the cross-section of the phase shifter with graphene heaters. The temperature was fixed at 298 K for all solid boundaries, while a convective heat flux boundary condition was implemented for the gaseous boundaries.

The thermally induced refractive index change causes the wavelength tuning of TO switches. The wavelength shift corresponding to the refractive index could be expressed by [66]:

(1) $$\Delta \lambda =\lambda_{0}\;\frac{\Delta n_{eff}}{n_{g}}$$

where *λ_0_* is the free-space wavelength, Δ*n_eff_* represents the variation of effective refractive index, and *n_g_* is the modal group index. Based on the perturbation theory, the effective refractive index tuning ∆*n_eff_* should be given by a surface integral about temperature, electric field, and refractive index [67]:

(2) $$\Delta n_{eff}=\frac{∯\left(\frac{dn}{dT}\right)\Delta T\left(x,y\right)n\left(x,y\right)|E\left(x,y\right){|}^{2}dxdy}{∯n^{2}\left(x,y\right)|E\left(x,y\right){|}^{2}dxdy}$$

where *dn/dT* is the material thermo-optic coefficient. The TO coefficients are approximately 1.76 × 10^−4^ K^−1^ for silicon [68] and 1.3 × 10^−5^ K^−1^ for silicon dioxide [69] at 2 μm, respectively. Thus, if the static and dynamic response of the graphene-on-SOI phase shifter could be obtained by numerical simulation, we could explore the influence of design parameters on the performance of the graphene-based thermo-optic switches.

In this section, we focus on the analysis of MRR type TO switch since it could gain much higher *FOM* benefiting from the resonance effect. The critical factors for the graphene-on-SOI phase shifter are the cladding thickness (*h_cl_*), contact distance (*d_c_*), and the resistance of the heating region. The measured resistance of the graphene-based MRR resonators is 125 Ω. It contained resistance (*R_g_*) from the graphene heater and contact resistance (*R_c_*) between metal and graphene. Considering the sheet resistance of graphene is 1307 Ω/sq, *R_c_* would be 4550 Ω·μm. Thus, the divided voltage *U_d_* applied on the graphene heater in the simulation is equal to *U∙R_g_/(R_t_ + R_c_),* where *U* is applied voltage across the electrodes. For a given length of graphene heater, the contact resistance would keep the same while distance between the two electrodes varied. Figure 5b plots the influence of cladding thickness (*h_cl_*) and contact distance (*d_c_*) on the temperature of the waveguide core. Although the increase of the contact distance *d_c_* would entail an increased divided voltage *U_d_* on the graphene heater, the increase of total resistance and thermal mass would lead to a decrease in temperature change Δ*T_wg_(x,y)* in the waveguide with the increasing contact distance, shown in Figure 5b. This is why the performance of our MZI type TO switch was worse than that of the MRR type TO switch since the contact distance is much larger. Besides, when the thickness of oxide cladding *h_cl_* becomes thicker, the graphene heater will keep further away from the waveguide core. The Δ*T_wg_(x,y)* will also decrease. For an MRR type graphene-based TO switch with a radius *r* of 40 μm and a heater length *l_h_* of 100 μm, the overall wavelength shifting could be expressed as follows:

(3) $$\Delta \lambda =\lambda_{0}\frac{\Delta n_{eff}}{n_{g}}\frac{I_{h}}{2\pi r}$$

Based on Equations (2) and (3), we can convert the thermal profile to the wavelength shift Δ*λ* profile, which is illustrated in Figure 5c. The heating efficiency *η* of switches with variable design parameters could be calculated by

(4) $$\eta =\frac{\Delta \lambda }{\Delta P}$$

where ∆*P* represents the change of electric power. The electric power can be calculated by *U^2^/R_t_*, where *R_t_* is equal to *R_g_ + R_c_*.

Figure 5d plots the heating efficiency of MRR type graphene-based TO switch with different cladding thicknesses and contact distances. The figure marked our measured device’s experimental (Exp) and simulated (Sim) heating efficiencies. The numerical analysis agrees pretty well with our measurement results. To be noted, only part of the applied voltage was on the graphene heater region. The heater’s efficiency could be further improved by minimizing the contact resistance, which will be discussed later.

The response time of the thermo-optic switch is significant for high-speed light intensity modulation and optical routing. Finite element method (FEM) simulation could also be applied to study the dynamic heating progress. The temporal evolution of the temperature of the waveguide core was simulated under a square-wave driving bias with a period of 30 μs, and the time-domain response progress of devices with different *h_cl_* and *d_c_* are presented in Figure 5d. The calculated response times for different device parameters are plotted in Figure 5e. Both the heating and thermal dissipation processes require a longer time for a larger heating region (thermal mass), and thus smaller *h_cl_* and *d_c_* are also desired for faster operation. The simulated response time is 3.735 μs which is very close to the experimental results of 3.96 μs.

**Figure 6:**
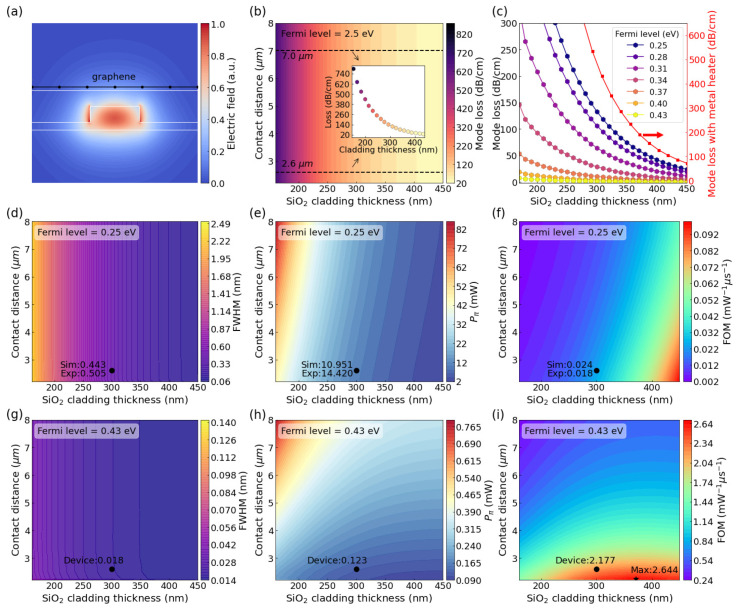
Simulation of the graphene-on-SOI structure. (**a**) Electric field distribution of the fundamental TE mode. (**b**) Mode loss of the fundamental TE mode versus *h_cl_* and *d_c_* with *E_F_* of 0.25 eV. (**c**) Mode loss versus *h_cl_* with different *E_F_* and fixed contact distance of 2.6 μm. The red curve denotes the mode loss of the waveguide with the metal heater. (**d**) *FWHM*, (**e**) power consumption *P_π_* and (**f**) *FOM* mapping for the MRR device with different *h_cl_* and *d_c_*, and Fermi level of 0.25 eV. (**g**) *FWHM*, (**h**) power consumption *P_π_* and (**i**) *FOM* mapping for the MRR device with different *h_cl_* and *d_c_*, and *E_F_* of 0.43 eV. Simulated (Sim) and experimental (Exp) values are labeled in (**d**–**f**). Simulated values of our devices (Device) are marked in (**g**–**i**). Star marker in (**i**) points out optimal structural parameters with maximum (Max) *FOM*.

Here, we can notice that TO switches could achieve a higher heating efficiency and faster response if the distance between the graphene and the waveguide and the distance between the two electrodes are close enough. However, the overall performance of TO switches will also be limited by the absorption from graphene heaters and the metal electrodes. Although metal absorption is much larger than graphene, graphene is still a lossy material if its Fermi level is low. Figure 6a illustrates the modal profile in the cross-section of the phase shifter with graphene heaters. Based on our measurement result, the graphene-induced total loss of the ring is 54.14 dB/cm, and the corresponding Fermi-level (*E_F_*) of graphene was 0.25 eV. Based on this Fermi-level, we studied the performance matrix of MRR type TO switches with different design parameters, as depicted in Figure 6b. It is illustrated that the optical loss induced by the graphene for the integrated ridge waveguide decreases exponentially as the cladding thickness increases. The inset shows the losses versus *h_cl_* with *d_c_* being 2.6 μm and 7 μm, respectively, and the propagation loss from the metal contact could be neglected if the distance between two electrodes is larger than 2.5 μm. Thicker cladding is preferred for less propagation loss, but it will sacrifice the heating effect. Thus, if we want to design an optimal heater structure with graphene, a compromise should be made between the heater loss and heating efficiency. 

For the ring resonator, the loss directly impacts the *Q* factor and the *FWHM* of the resonance peaks and consequently the power consumption. The power consumption for *P_π_ = FWHM×π/η* and the relation between the total *Q* factor and the *FWHM* is expressed by

(5) $$Q_{tot}=\frac{\lambda_{r}}{FWHM}$$

where *λ_r_* is the resonance wavelength. To obtain the best performance, the ring resonator should work in the critical coupling condition where the intrinsic *Q* factor equals the external *Q* factor:

(6) $$\frac{1}{Q_{tot}}=\frac{1}{Q_{intrinsic}}+\frac{1}{Q_{external}}=\frac{2}{Q_{intrinsic}}$$

The intrinsic *Q* factor can be obtained by

(7) $$Q_{intrinsic}=\frac{2\pi n_{g}}{\alpha \lambda_{r}}$$

where *α* is the loss of the ring, including the absorption of the graphene *α_g_* and the waveguide loss *α_wg_* (1.62 dB/cm). The *n_g_* is the group index of the fundamental TE mode. The loss of the resonator is expressed by:

(8) $$\alpha =\alpha_{g}\frac{I_{h}}{2\pi r}+\alpha_{wg}$$

Therefore, based on Equations (5)–(8) [66], and the modal loss plotted in Figure 6b, the *FWHM* of resonator covered by graphene with the same Fermi level (0.25 eV) and length as our device’s with different *h_cl_* and *d_c_* can be obtained and depicted in Figure 6d. In the calculation, *n_g_* is 3.68 and *λ_r_* is 2022.74 nm. Thicker cladding leads to a narrower *FWHM*, implying that a smaller wavelength shift is necessary to achieve effective modulation. Based on the *FWHM,* combined with the efficiency *η*, power consumption *P_π_* was calculated and shown in Figure 6e. Finally, the *FOM,* which is equal to 1/(*P_π_·τ*), could be obtained, and they are plotted in Figure 6f. We have marked the experimental and numerical simulation predicted device performance results in all three figures. All simulated performance indices are in good agreement with the experimental results, indicating the reliability of our models.

As discussed before, the loss induced by graphene could significantly influence the *FWHM* of the resonance peak and the *FOM* of MRR type TO switches. Fortunately, the loss of the graphene decreases significantly with the doping level of graphene (*E_F_*), as shown in Figure 6c. To investigate the performance limit of the TO switch with a graphene heater, we performed calculations based on heavier doping of graphene, i.e., *E_F_* = 0.43 eV (which is achievable [70]). Thus, the optical loss induced by graphene could be decreased a lot. The quality factor could be much higher, and the *FWHM* could be an order of magnitude smaller than those based on graphene with a Fermi-level of 0.25 eV, as shown in Figure 6g. Besides, the contact resistivity could be optimized below 100 Ω μm [71], and the contact resistance could be less than 1 Ω while currently it is approximately 45.5 Ω. Thus, most of the applied voltage is on the graphene heater, and the power efficiency could be much higher. Under these conditions, the *FOM* can be distinctly boosted. *P_π_* and *FOM* were calculated and demonstrated in Figure 6h,i. From Figure 6i, the highest *FOM* (2.644 mW^−1^μs^−1^) could be achieved with a 371-nm cladding thickness and 2.25-μm contact distance.

As mentioned, the heavily p-doped graphene heater owns obviously smaller absorption loss, and therefore the minimum *FOM* in Figure 6i, i.e., 0.217 mW^−1^μs^−1^ is already an ultrahigh value. Hence, graphene TO devices would allow much larger fabrication-error tolerance to achieve high performance than other technologies. Relatively high *FOM* can be obtained with thin cladding, and the current device design can achieve an extremely high *FOM* of 2.177 mW^−1^μs^−1^.

Using these models, the *FOM* of a switch with a metal heater could also be evaluated. The red curve in Figure 6c is the calculated modal loss of waveguide with a metal heater above, exhibiting absorption at least two orders of magnitude greater than those with graphene even with high Fermi levels (0.25 eV). The *FOM* of a 50-nm-thick gold heater MRR TO switch with the same cladding thickness and contact distance is only 0.005 mW^−1^μs^−1^, which is three orders of magnitude less than that with graphene heaters. As known, the absorption coefficient for the metal increases with the wavelength. Thus, metal heater should be placed much further away from the waveguide core in the mid-infrared range. On the other hand, graphene could become transparent in the mid-infrared wavelength range through tuning its Fermi level. Thus, the *FOM* of graphene-based TO switches could be much higher than those based on metal heaters. Graphene will become an exceptional nanomaterial for future MIR photonic circuits.

## 5. Conclusions

We demonstrated MRR-based and MZI-based TO switches operating at 2 μm waveband for the first time, where single-layer graphene was adopted as the heater. For the MRR device, the modulation efficiency was 0.11 nm/mW, corresponding to minimum power consumption of about 5 mW. The 10–90% rise time/90–10% fall time is 3.7/3.96 μs. Such dynamic response is close to the fastest silicon TO devices ever reported. As for the MZI-based device, an insertion loss of 2.0 dB and an outstanding extinction ratio of 27.08 dB with broadband operation wavelength range was achieved. The switching time was also characterized to be 4.92/4.97 μs respectively. The insertion loss could be minimized by the chemical doping of graphene, and the relatively high power consumption can be largely suppressed by a more appropriate design of contact distance. The numerical analysis showed that the performance of the graphene-assisted TO switch can be improved significantly by appropriately designing the structure of the heater and doping the graphene. The *FOM* can be as high as 2.644 mW^−1^μs^−1^, which outclasses reported works. The doping engineering can make graphene an almost transparent heater in MIR waveband, which can be directly placed on the waveguide. Such unparalleled performance can hardly be achieved by resorting to metal heaters or doped silicon heaters. Our work proves that graphene heater owns exceptional advantages compared to other kinds of heaters, and graphene is a superb photonic material for future photonic integrated circuits working at MIR waveband.

## Figures and Tables

**Table 1:** Performance comparison of some silicon TO devices working at 2 μm.

Device	Heater	*η* (nm/mW)	*τ* (μs) Rise/Fall	*P_π_* (mW)	*FOM * (mW^−1^μs^−1^)	Year [Ref.]
MZI	TiN	N/A	15/15	32.3	0.002	2019 [32]
MZI	TiN	N/A	9.2/13.2	19.2	0.004	2021 [33]
MZI	Doped silicon	0.17	3.49/3.46	25.21	0.011	2021 [36]
MRR	Doped silicon	0.1	3.65/3.70	6.66	0.0405	2021 [36]
MRR	graphene	0.11	3.72/3.96	14.42	0.0175	this work
MZI	graphene	N/A	4.92/4.97	57.75	0.003	this work
MRR	graphene	0.127	3.735/-	0.123	2.644	Prediction
